# In Search of the Active Metabolites of an Anticancer Piperazinedione, TW01003, in Rats

**DOI:** 10.1155/2014/793504

**Published:** 2014-04-17

**Authors:** Chun-Li Wang, Ching-Kuei Chen, Yao-Horng Wang, Yu-Wen Cheng

**Affiliations:** ^1^School of Pharmacy, College of Pharmacy, Taipei Medical University, No. 250, Wu-Hsing Street, Taipei 110, Taiwan; ^2^Research and Development Center, United Biomedical, Inc., Asia, No. 45, Guangfu N. Road, Hukou, Hsinchu 303, Taiwan; ^3^Department of Nursing, Yuanpei University, No. 306, Yuanpei Street, Xiangshan District, Hsinchu 300, Taiwan

## Abstract

TW01003, a piperazinedione derivative designed as an antimitotic agent, exhibited potent anticancer and antiangiogenesis activities in mice. However, oral administration of this compound in rats led to poor systemic bioavailability which suggested that *in vivo* efficacy might come from its metabolites. This report describes the identification of TW01003 metabolites in pig and Wistar rats. Following intravenous administration of TW01003, pig urine samples were subjected to sulfatase and glucuronidase treatment to monitor the biotransformation products. Rats were given TW01003 both intravenously and orally, and blood samples were collected and then analyzed by HPLC to quantitatively determine the metabolic transformation of TW01003 to its metabolite. A sulfate conjugate, TW01003 sulfate, was identified as the major metabolite for TW01003 after intravenous injection in both pig and rats. However, in rats, the glucuronide conjugate became major metabolite 30 min after TW01003 oral dosing. Pharmacokinetic analysis after intravenous administration of TW01003 indicated that TW01003 sulfate had a systemic bioavailability 2.5 times higher, volume of distribution three times higher, residence time seven times longer, and clearance rate 2.3 times lower compared to TW01003. Our results indicate that the potent anticancer and antiangiogenesis activities of TW01003 might not come from TW01003 *per se* but from its metabolites TW01003 sulfate.

## 1. Introduction

**Figure 1:**
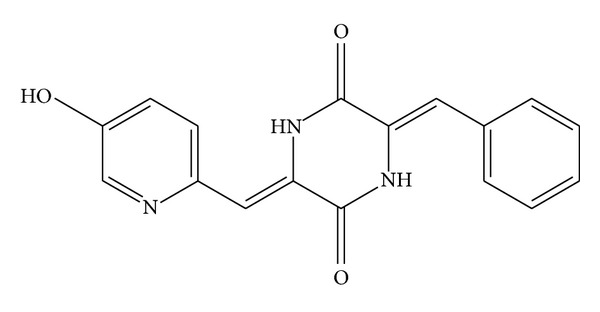
Structure of TW01003 in mice.

Tubulin binding agents, which result in mitotic arrest of tumor cell and then apoptosis, are now standard treatment in cancer chemotherapy [1–4]. (3E,6E)-3-Benzylidene-6-[(5-hydroxypyridin-2-yl)methylene]piperazine-2,5-dione (TW01003) (Figure 1), a piperazinedione derivative synthesized in this laboratory as an antimitotic agent, exhibited a broad spectrum of antitumor activities in 60 human disease-oriented cancer cell panel screenings [5–7]. A profound antiangiogenesis effect of this compound was demonstrated in mice; after oral treatment of TW01003 (3 mg/kg), the hemoglobin count of the matrigel with vascular endothelial growth factor- (VEGF-) induced angiogenesis was reduced to <1% (unpublished data). However, a preliminary pharmacokinetic study indicated poor bioavailability upon oral administration of this compound to rats, with only 1.72% of the oral fraction absorbed (unpublished data). This led to a suspicion that the potent antiangiogenesis effect of TW01003 might come from its metabolites.

In this report, we describe the identification of the metabolites of TW01003 in pig following intravenous (i.v.) administration. We also investigated the metabolic profiles of TW01003 in rats following i.v. and oral administration. The transformation of TW01003 to its major metabolite was determined by pharmacokinetic studies in rats.

## 2. Materials and Methods

### 2.1. Materials

TW01003 potassium salt (TW01003-K) was prepared in this laboratory [5]. Analytical grade chemicals for biological studies were from Sigma-Aldrich (St. Louis, MO, USA), E. Merck KG (Darmstadt, Germany), Fluka Chemika (Buchs, Switzerland), Acros (Morris Plains, NJ, USA), and Wako (Richmond, VA, USA). High-performance liquid chromatography- (HPLC-) grade acetonitrile and methanol were purchased from Alpus Pharmaceutical Industries Co. (Gifu, Japan). Equipment used in the preparation of biological samples consisted of the following: Branson Sonifier 450 sonicator (Danbury, CT, USA), Kubota 2010 (Tokyo, Japan), Eppendorf AG 5415C centrifuge (Hamburg, Germany), and Model 905 incubator (Ballrechten-Dottingen, Germany). *β*-Glucuronidase (type B-1 from bovine liver, containing 1,240,000 units/g of *β*-glucuronidase) and sulfatase (type H-1 from* Helix pomatia*, containing 14,000 units/g of sulfatase and 498,800 units/g of *β*-glucuronidase) were purchased from Sigma-Aldrich.

Crossbred 3-4-month-old pigs weighing 40–50 kg, obtained from the Animal Technology Institute, Taiwan (Miaoli, Taiwan), were used in a preliminary metabolic study for establishing the HPLC analytical method for the identification of TW01003 and its metabolites. The phase II conjugate TW01003 sulfate was first isolated from pig urine as an authentic sample for analysis. Male Wistar rats (200–250 g) for pharmacokinetic studies were purchased from the Laboratory Animal Center of National Taiwan University (Taipei, Taiwan). The animals were pathogen free and allowed to acclimate to the environmentally controlled quarters (24 ± 1°C and 12 : 12 h light-dark cycle) for at least 5 days before the experiments. Animal studies were conducted in accordance with the Guide for the Care and Use of Laboratory Animals [8].

### 2.2. Analytical Sample Preparation

D(+)-Glucose-monohydrate (D5W) solution (5%) was prepared by mixing 25 g of D5W with double deionized water, filtered through a 0.22 *μ*m Millipore filter, and adjusted to 500 mL. Vehicle V was prepared by mixing 5% D5W, Cremophor EL, and ethyl alcohol in the ratio of 90 : 5 : 5 (v/v/v). Standard TW01003 solution (1 mg/mL) was prepared by dissolving TW01003-K (1.16 mg) in 1 mL of vehicle V and 160 *μ*L of aqueous 25% NH_4_OH solution to become a stock solution with a concentration of 1 mg/mL. The solution was stored at 4°C until use.

The stock solution was diluted with vehicle V to prepare standard solutions with concentrations ranging from 4.0 *μ*g/mL to 0.00781 *μ*g/mL. The TW01003 standard solutions (100 *μ*L) were mixed with blank plasma (100 *μ*L) and an internal standard solution (400 *μ*L of a solution of HPW044X11 in ethyl acetate 0.025 *μ*g/mL) and centrifuged (5,585 g) for 10 min. The supernatant was blown to dryness with nitrogen gas. The D5W vehicle (200 *μ*L) was then added to the residue as the test solution, and 100 *μ*L was subjected to HPLC analysis.

### 2.3. Chromatography and Validation of Assay Methods

TW01003 test solutions were analyzed by HPLC. The HPLC system consisted of an autosampler (AS950, Jasco, Tokyo, Japan), a Waters Model 600E solvent delivery pump (Millipore, Milford, MA, USA) coupled with an ultraviolet detector monitored at wavelength 350 nm (Bioanalytical Systems, Inc., West Lafayette, IN, USA), and an integrator (Macintosh LC II computer with Macintegrator I, Rainin; Apple, New York, NY, USA).

The samples were eluted in a C18 reversed-phase microbore column (particle size 5 *μ*m, 150 × 1 mm; Bioanalytical Systems) at a flow rate of 1 mL/min. The eluent was filtered through a Millipore filter (0.22 *μ*m) and degassed prior to analysis. Mobile phases for gradient elution were 0.1% acetic acid aqueous solution (HOAc_(aq)_) : acetonitrile (ACN) = 60 : 40 at 0–10 min and 0.1% HOAc_(aq)_ : ACN = 50 : 50 at 10–23 min.

Assay methods were validated by determining the precision and accuracy of intra- and interday analyses of serum standards over a period of 6 days. The lower limit of quantification (LOQ) between intraday assays of TW01003 was 7.46 ± 0.67 ng/mL (*n* = 3, *r* ^2^ = 0.9995), with coefficients of variation less than 9%. The lower LOQ between interday assays was 7.17 ± 0.54 ng/mL (*n* = 3, *r* ^2^ = 0.9999) with coefficients of variation less than 15%.

### 2.4. Animal Experiments

Single-dose TW01003 in a solution (1 mL) containing a 9 : 1 (v/v) ratio of vehicle V : 10% Na_2_CO_3(aq)_ was used for animal studies in pig or in male Wistar rats [9]. For metabolic studies, the TW01003 test solution was administered intravenously (7 mg/kg, *n* = 1) to the tail vein or orally (36 mg/kg, *n* = 1) by a feeding tube. For pharmacokinetic studies, the test solution was administered intravenously to the tail vein of Wistar rats (2.0 mg/kg, 1 mL, *n* = 6). The rats were put under a heating lamp to maintain body temperatures at 37°C throughout the experiment. All procedures involving the use of animals were approved by the Institutional Animal Care and Use Committee of Taipei Medical University.

Blood samples (0.5 mL) were withdrawn from the carotid artery of rats at time intervals of 5, 15, 30, 45, 60, 90, 120, 180, and 240 min. Heparin sodium (25 IU/mL in 0.3 mL of saline) was added, and the blood samples were centrifuged (5,585 g) at 4°C for 8 min. The plasma was frozen immediately and kept at −78°C until analysis. The plasma sample (150 *μ*L) was mixed with a sulfatase solution (200 IU/mL, 50 *μ*L) at 37°C for 2 h. The plasma sample (150 *μ*L) mixed with 50 *μ*L of a buffer solution (pH 5.0) was used for the control group. The internal standard solution was then added, and the solution was centrifuged (5,585 g) for 10 min. The supernatant was concentrated to dryness with nitrogen gas. The D5W vehicle (200 *μ*L) was then added to the residue, and the solution was subjected to HPLC analysis.

### 2.5. Pharmacokinetic Studies

We established the plasma concentration-time profile [10, 11]. The area under the plasma concentration-time profile (AUC) and other pharmacokinetic parameters (peak concentration: *C*_max⁡_; time to reach *C*_max⁡_: *T*_max⁡_; area under the moment curve: AUMC; volume of distribution: *V*_*D*_) were calculated using the log-linear trapezoidal rule. Plasma concentrations of TW01003 and the major metabolite were calculated using WINNONLIN 3.1 software by a noncompartment model. Terminal half-life (*t*_1/2_) was compartment model-independently estimated. Data analysis was performed using Microsoft Excel, and data were represented as mean ± standard deviation (SD) for *n* experiments. Treatment differences were evaluated by the paired *t*-test.

## 3. Results

### 3.1. Chromatographic Identification of TW01003 and TW01003 Sulfate

**Figure 2:**
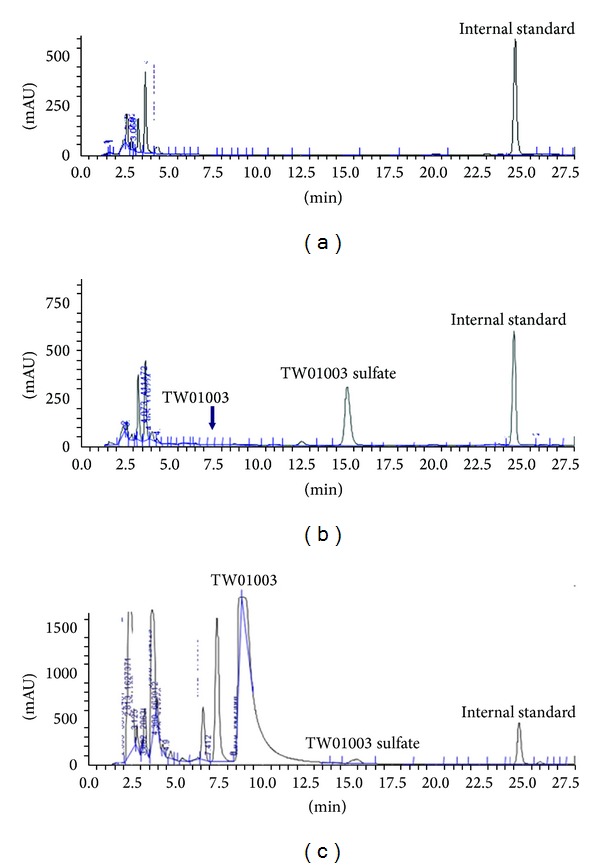
Typical high pressure liquid chromatograph of untreated urine of pig (a); pig urine after TW01003 administration (b); and sulfatase-treated pig urine after TW01003 dosing (c). Parent compound TW01003, TW01003 sulfate, and internal standard HPW044X11 were identified with retention times at 9.17 min, 16.59 min, and 25.85 min, respectively. TW01003 glucuronide with a retention time at 12.5 min was barely visible.

In order to establish a feasible analytical method for the identification of TW01003 metabolites in a biological system, a test solution containing 200 mg of TW01003 was first injected into a 40–50 kg pig. Fresh urine (1 mL) was collected 60 min after dosing, filtered through a 0.22 *μ*m Millipore filter, and subjected to HPLC analysis. Typical HPLC chromatograms of urine sample of untreated pig (Figure 2(a)), urine samples upon i.v. dosing of TW01003 (Figure 2(b)), and urine samples after sulfatase treatment (Figure 2(c)) are depicted.

### 3.2. Metabolic Study of TW01003 in Rats

**Figure 3:**
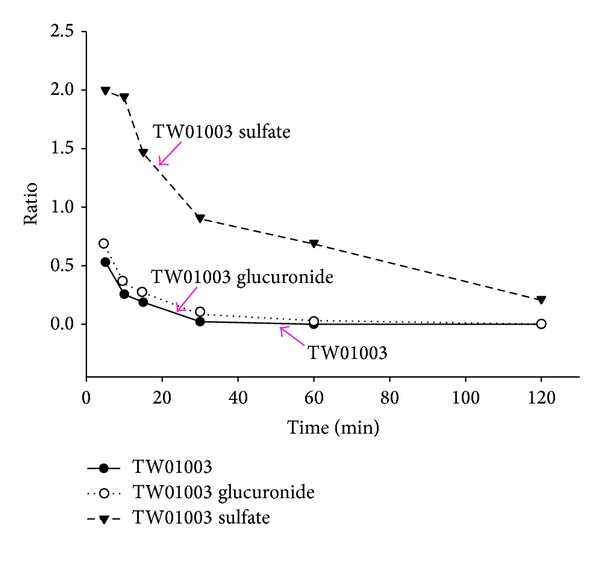
Plasma concentration-time curve after i.v. administration of TW01003 (6.7 mg/kg) to a rat. Plasma samples were collected and subjected to enzymatic treatment by sulfatase or glucuronidase. This method was used for the identification of TW01003 sulfate or glucuronide conjugate after TW01003 dosing.

**Figure 4:**
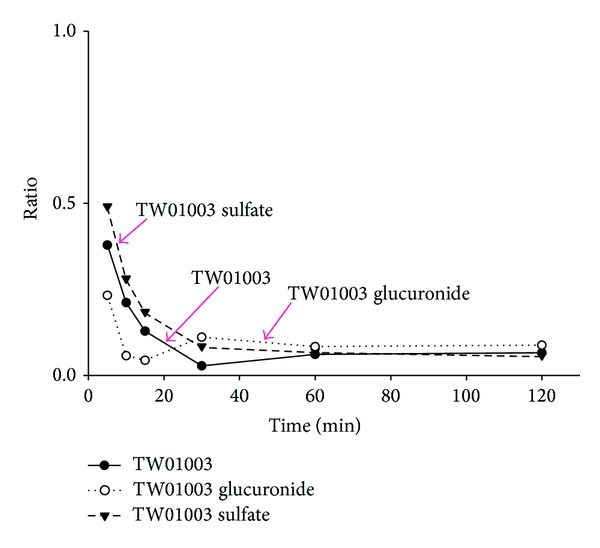
Plasma concentration-time curves of TW01003 and its metabolites after oral administration of TW01003 (36 mg/kg) to a rat (*n* = 1).

Metabolic studies of TW01003 were conducted using male Wistar rats. The preliminary data were studies in one rat. In this experiment, we had established plasma concentration-time curves of parent TW01003 and its major metabolites after i.v. (6.7 mg/kg, *n* = 1, Figure 3) or oral administration (36.0 mg/kg, *n* = 1, Figure 4) of TW01003. Plasma samples were collected and subjected to enzymatic treatment by sulfatase or glucuronidase for the identification of TW01003 sulfate and glucuronide as phase II metabolic conjugates.

The results indicated that TW01003 was cleared fairly rapidly, regardless of administration method. As sulfonation and glucuronidation are the most common phase II metabolic pathways for compounds containing an aromatic hydroxyl group, we first identified the metabolites in rat urine. Urine samples were subjected to sulfatase or glucuronidase treatment prior to HPLC analysis. The method used for analyze TW01003 was also used to identify TW01003 sulfate or glucuronidase conjugate.

### 3.3. Pharmacokinetics upon I.V. Administration of TW01003 in Rats

**Figure 5:**
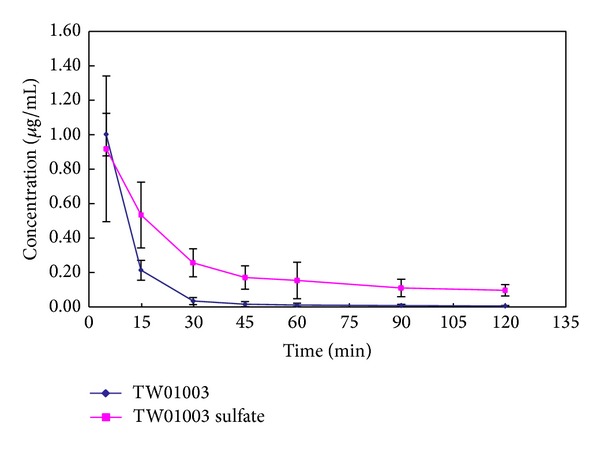
Plasma concentration-time profile of TW01003 and TW01003 sulfate after i.v. administration of TW01003 (*n* = 6).

Plasma samples were subjected to sulfatase treatment for the identification of TW01003 sulfate conjugate as a metabolite. The plasma concentration-time profile of TW01003 and TW01003 sulfate after i.v. administration of TW01003 is depicted in Figure 5 (*n* = 6). Pharmacokinetic (PK) parameters of TW01003 and TW01003 sulfate are summarized in Table 1.

## 4. Discussion

This study investigated the biotransformation of TW01003 in rats and in pig. As depicted in Figure 2(b), we observed a fast clearance upon administration of TW01003 in pig. After 60 min of TW01003 dosing, TW01003 sulfate (retention time 16.59 min) was identified as the major metabolite. Upon sulfatase treatment, this phase II conjugate was hydrolyzed to TW01003 (Figure 2(c)), confirming that the peak of retention time 16.59 min is TW01003 sulfate conjugate. We had also purified the TW01003 sulfate from pig urine, which showed the same retention time in HPLC (data not shown). This enzymatic method was used to quantify the biotransformation process of TW01003 biotransformation to TW01003 sulfate.

We conducted pharmacokinetic studies to monitor the blood level of TW01003 and TW01003 sulfate. In Wistar rats (Figure 5, *n* = 6), the pharmacokinetic parameters of TW01003 and TW01003 sulfate after i.v. administration of TW01003 were derived from noncompartmental model, based on plasma concentration-time profile. The systemic exposure of TW01003 sulfate was 2.5 times higher than that of TW01003 (AUC from time 0 to infinite, AUC_INF_  0.71 ± 0.25 versus 0.29 ± 0.04 h·*μ*g/mL). Interestingly, TW01003 sulfate demonstrated a three times higher volume of distribution (*V*_*D*_  4.43 ± 1.52 versus 1.41 ± 0.41 L/kg), seven times longer residence time (MRT_INF_  1.44 ± 0.33 versus 0.20 ± 0.06 h), and 2.3 times lower clearance rate (CL 3.08 ± 0.90 versus 7.05 ± 1.12 L/h/kg) than those of TW01003.

Sulfonation and glucuronidation are the most common metabolic pathways of compounds with aromatic hydroxyl groups [12–14]. This kind of phase II metabolism is also demonstrated in TW01003. The plasma concentration-time profile after i.v. administration of TW01003 to Wistar rats demonstrated a fast metabolism of this compound to its sulfate conjugate as the major metabolite (Figure 3). However, the glucuronide conjugate became the major metabolite 30 min after TW01003 oral dosing (Figure 4). Enterohepatic circulation might explain the increased bioavailability of this metabolite in the systemic circulation. The difference in the sulfonation product level after i.v. or oral dosing of TW01003 may have resulted from tissue distribution of the sulfonation enzyme sulfotransferase [15, 16].

**Figure 6:**
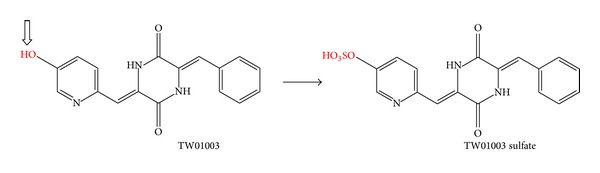
Biotransformation of TW01003 to TW01003 sulfate.

Sulfotransferase, a family of enzymes encoded by sulfotransferase (SULT) genes, possesses important physiological functions in the regulation of bile acid enterohepatic circulation [17]. It also transfers a sulfate group from 3′-phosphoadenosine 5′-phosphosulfate to xenobiotics, primarily to aromatic alcohol, to enhance the hydrophilicity of its substrate and thus improve the clearance of the foreign molecules through bile or renal secretion. From the chemical structure of TW01003 shown in Figure 6, the only viable aromatic hydroxyl group is on the pyridine ring, which is not commonly seen in biotransformation reactions [18].

There were various mutations identified in the SULT enzyme family and they were reported to exhibit different catalytic specificity and capacity in each polymorph form [15]. For new drug development, identification of the metabolic pathway is essential. More studies are needed to explore the enzyme system involved in the sulfonation process.

TW01003 is an antiangiogenesis agent that exhibits strong potency in an* in vivo* model. However, the limited half-life failed to explain its* in vivo* efficacy. Sulfonation in metabolism is mainly regarded as a detoxification mechanism. It may also serve as a bioactivation process [18, 19] that can activate TW01003 with improved exposure, longer half-life, and longer mean residence time. Further investigations are needed to confirm the antiangiogenesis activity of TW01003 sulfate metabolites.

## 5. Conclusion

Administration of TW01003 exhibited extensive metabolism in both pig and rats. Extensive metabolism leading to the high clearance rate of TW01003 in this study explains the unsatisfactory pharmacokinetics of TW01003. As TW01003 exhibited potent anticancer and antiangiogenesis activities, the low systemic bioavailability of TW01003 failed to explain the fact that the activity came from TW01003* per se*. From the chemical structure of TW01003, we suggested an unusual pyridine-OH sulfonation metabolite, and this may serve as an active metabolite for its antiangiogenesis potency. Further studies are needed to identify if the metabolites that demonstrated the anticancer and antiangiogenesis activities are active species.

## Figures and Tables

**Table 1:** Summary of pharmacokinetic parameters of TW01003 and TW01003 sulfate after i.v. administration of TW01003 (*n* = 6).

PK parameters	TW01003	TW01003 sulfate
	Mean ± SD	Mean ± SD
*C*_ max_ (*μ*g/mL)	2.20 ± 0.25	1.31 ± 0.64
*t*_ 1/2_ (h)	0.76 ± 0.14	1.31 ± 0.22
AUC_all_ (h∗*μ*g/mL)	0.28 ± 0.04	0.52 ± 0.20
AUC_INF_ (h∗*μ*g/mL)	0.29 ± 0.04	0.71 ± 0.25
CL (L/h/kg)	7.05 ± 1.12	3.08 ± 0.90
AUMC_INF_ (h∗h∗*μ*g/mL)	0.06 ± 0.02	1.01 ± 0.36
MRT_INF_ (h)	0.20 ± 0.06	1.44 ± 0.33
*V*_*D*_ (L/kg)	1.41 ± 0.41	4.43 ± 1.52

*C*_ max_: peak concentration; *t*_1/2_: half-life; AUC_all_: area under curve from time 0 to the last sampling time; AUC_INF_: area under curve from time 0 to infinite; CL: clearance; AUMC_INF_: area under the moment-time curve from time 0 to infinite; MRT_INF_: mean residence time from time 0 to infinite; *V*_*D*_: volume of distribution.
